# Olfactory Drug Aerosol Delivery with Acoustic Radiation

**DOI:** 10.3390/biomedicines10061347

**Published:** 2022-06-08

**Authors:** Mohammad Yaghoub Abdollahzadeh Jamalabadi, Jinxiang Xi

**Affiliations:** Department of Biomedical Engineering, University of Massachusetts, Lowell, MA 01854, USA; muhammad_yaghoob@yahoo.com

**Keywords:** direct nose-to-brain delivery, olfactory deposition, active particle control, acoustic radiation

## Abstract

Nose-to-brain (N2B) drug delivery is a new approach to neurological disorder therapy as medications can bypass the blood-brain barrier and directly enter the brain. However, the delivery efficiency to the olfactory region using the conventional delivery method is impractically low because of the region’s secluded position in a convoluted nasal cavity. In this study, the acoustic radiation force was explored as an N2B delivery alternative in a wide frequency range of 10–100,000 Hz at an increment of 50 Hz. Numerical simulations of the particle deposition in the olfactory region of four nasal configurations were performed using COMSOL. Frequency analysis of the nasal cavities revealed that eigenfrequencies were often associated with a specific region with narrow passages and some eigenfrequencies exhibited an amendable pressure field to the olfactory region. Transient particle tracking was conducted with an acoustic inlet at 1 Pa, and a frequency spectrum of 10–100,000 Hz was imposed on the airflow, which carried the particles with acoustic radiation forces. It was observed that by increasing the pulsating wave frequency at the nostrils, the olfactory delivery efficiency reached a maximum in the range 11–15 kHz and decreased after that. The correlation of the olfactory delivery efficiency and instantaneous values of other parameters such as acoustic velocity and pressure in the frequency domain was examined.

## 1. Introduction

**Figure 1:**
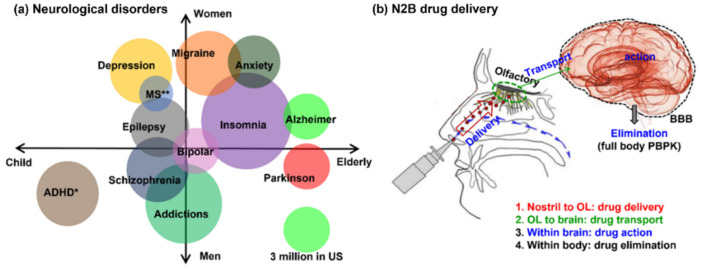
Prevalence and treatment of neurological disorders (NDs): (**a**) various types of NDs and their prevalence (scale: the green circle representing 3 million patients), and (**b**) four stages of nose-to-brain (N2B) drug delivery: (1) intranasal drug delivery to the olfactory mucosa, (2) drug transport from the olfactory mucosa to the brain (tissue transport), (3) drug action in the brain (pharmacodynamics), and (4) drug elimination from the brain and body.

There are more than 600 types of neurological disorders (NDs), which afflict more than 30 million individuals and incur tremendous medical costs [1]. The ND incidence is expected to continue rising due to a longer life span [2]. NDs are not limited to Alzheimer’s and Parkinson’s diseases that are more prevalent in senior populations; they also occur in children, such as attention deficit hyperactivity disorder (ADHD) and autism [3,4,5] (Figure 1a). Some NDs are gender-specific; depression, migraine, and anxiety are more often seen in females, while addictions are more often seen in males. Some NDs can occur at all ages and for both genders, such as bipolar, insomnia, epilepsy, and multiple sclerosis [6].

Due to the challenges from the blood-brain barrier (BBB), many efforts have been undertaken to explore the intranasal route to deliver medications directly to the brain, termed direct nose-to-brain (N2B) drug delivery [7]. Intranasal drug delivery provides a noninvasive practical method to bypass the BBB and directly deliver the medications to the brain and spinal cord. This method works because of the unique connection that the olfactory nerves provide between the brain and the environment. Intranasally administered drugs can reach the central nervous system (CNS) via the olfactory and trigeminal extracellular pathways within minutes, resulting in quick therapeutic onset [8]. A growing number of recent reports have demonstrated the effectiveness of intranasal administration of neurotherapeutic agents to the brain [9,10]. Thus, many drugs that have been previously abandoned due to the BBB may be worthy of being re-examined for intranasal delivery, thereby expanding the horizon of candidate agents. Currently, some therapeutic agents are undergoing clinical trials utilizing nose-to-brain delivery, such as neurotrophins for Alzheimer’s diseases [11], insulin for diabetic neuropathy [12], antiretrovirals for NeuroAIDS [8], and deferoxamine for stroke [13].

A complete N2B delivery involves four stages: (1) intranasal drug delivery to the olfactory mucosa, (2) drug transport from the olfactory mucosa to the brain (tissue transport), (3) drug action in the brain (pharmacodynamics), and (4) drug elimination from the brain and body (Figure 1b). One of the first researches on the use of the olfactory path for neurological disorder treatment was conducted by researchers [14,15], who explored the intranasal route to send nerve growth factors to the brain (i.e., the second stage in Figure 1b). Since then, efforts to enhance the transportation of small interfering molecules to the brain have been actively pursued [16]. The use of nanoparticles for N2B delivery, such as solid lipid nanoparticles, elastic polymers, emulsions, and liposomes, also gained popularity in recent years due to their stability, mobility, and modifiability [17]. Studies of acoustic drug delivery of microcapsules were reviewed by Kooiman et al. [18], which showed that the resonance frequency of microcapsules intensified the interactions with the environment and maximized the streaming potential. Ultrasound was also explored to loosen the olfactory tight junctions to enhance drug delivery efficiency to the brain [19].

However, precise targeting of drugs to the olfactory region (i.e., the first stage in Figure 1b) has been proven to be highly challenging, as there is no linear pathway from the nostrils to this region [20,21]. With standard intranasal delivery devices, most medications targeting the olfactory region will be filtered out in the front nose and turbinates [22,23]. Previous numerical simulations indicated that less than 1% of inhaled aerosol could reach the olfactory region [24]. The extremely low olfactory dosage emphasizes the need to develop novel strategies that can deposit drugs in the olfactory region with potent and repeatable doses.

There are some statistical methods to evaluate the drug delivery from nasal deposition results [25,26]. In the literature, researchers report the nasal deposition of particles in a range with the maximum olfactory deposition fraction [27]. As well there are some recent published papers for enhancing in vitro drug delivery in a model of the nasal cavity by radiating force [28,29,30,31]. However, the effect of excitation frequency in a large extent is not examined yet.

An improved way of targeting medications to the olfactory region is desired so that optimal therapeutic outcomes can be achieved, and adverse side effects can be minimized. The objective of this study is to numerically quantify the olfactory deposition fractions with acoustic streaming in various nasal model geometries. In this study, instead of design of a penetrating BBB model or CNS penetration, the focus is on the fact that the drugs for nasal cavity delivery are intended to rapidly enter the blood circulation. Specific aims include:

(1) to develop a computational model of olfactory drug delivery with acoustic radiation forces in four nasal geometries.
(2) to understand the acoustic responses of varying nasal geometries to pulsating waves from the nostrils.
(3) to predict the olfactory deposition fraction in the four nasal geometries under varying pulsating frequencies ranging from 10 Hz to 100,000 Hz.

The results of this study will provide insights into the dose enhancement that can be expected from acoustic-aided strategies for olfactory drug delivery.

## 2. Materials and Methods

### 2.1. Nasal Airway Models

**Figure 2:**
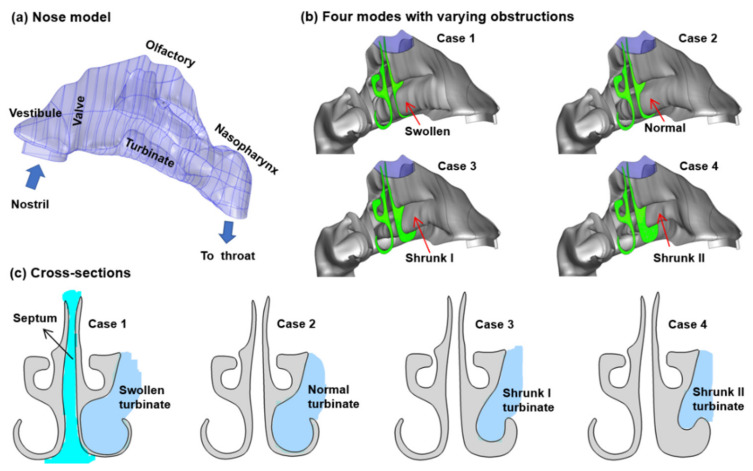
Computational models: (**a**) nose model geometry consisting of the nostril, vestibule, nasal valve, turbinate, nasopharynx, and the olfactory region, (**b**) four models with varying obstructions: Cases 1–4 with a progressively shrunk inferior turbinate, (**c**) cross-sections of Cases 1–4.

In this section, mathematical modeling of olfactory drug delivery in varying nasal geometries was thoroughly investigated. A schematic of a nasal cavity is depicted in Figure 2, which consists of different sections, such as the nostril, vestibule, nasal valve, turbinate, olfactory region, and nasopharynx (NP). The nasal model can be used for both numerical simulations and in vitro deposition experiments. This model (Case 2, or baseline) was subsequently modified in SolidWorks by either decreasing the dimension of the inferior meatus to generate Case 1, or increasing the inferior meatus dimension to generate Case 3 and Case 4 (Figure 2b).

In the baseline case (Case 2), the left and right turbinate sections were approximately symmetrical. However, symmetry is not the common phenomenon observed with typical in vivo conditions, considering the nasal cycle causes one turbinate to be consistently larger than its counterpart. The turbinate undergoes periodic swelling/deswelling that reduces or increases the flow area in the nasal passage. Therefore, various sizes of the turbinate can be observed throughout the day and must be incorporated into the model to provide proper airway dynamics that predict drug deposition. A cross-section through the turbinate of the four nasal geometries is compared in Figure 2c, which clearly exhibits a constricted meatus in Case 1 due to a swollen inferior turbinate, and a progressively enlarged meatus in Case 3 and Case 4 due to a shrunk turbinate.

### 2.2. Numerical Models

The governing equation for acoustic pressure in the nasal airway is shown below,

(1) $$\nabla \cdot \left(-\frac{1}{\rho_{c}}\left(\nabla p_{t}-q_{d}\right)\right)-\frac{k_{\mathrm{eq}}^{2}p_{t}}{\rho_{c}}=Q_{m},$$

where $p_{t}=p+p_{b}$ is the total pressure and $k_{\mathrm{eq}}^{2}={\left(\frac{\omega }{c_{c}}\right)}^{2}$. Monodisperse aerosols of 1-µm were tracked using the Lagrangian method as

(2) $$\frac{d\left(m_{p}v\right)}{dt}=F_{g}+F_{aco},$$

The acoustic force was calculated from the acoustic field

(3) $$F_{\mathrm{aco}}=-\nabla U_{\mathrm{rad}},$$

where the radiation field is

(4) $$U_{\mathrm{rad}}=v_{p}\left[f_{1}\frac{1}{2\rho c^{2}}\langle p^{2}\rangle -f_{2}\frac{3}{4}\rho \langle v_{\mathrm{in}\;}^{2}\rangle \right].$$

Here $f_{1}=1-\frac{K_{0}}{K_{p}}$ and $f_{2}=\frac{2\left(\rho_{p}-\rho \right)}{2\rho_{p}+\rho }$. The constant values of Equation (1) are presented in Table 1. Although the conditions, such as flow inlet temperature, and wall material, are not biologically relevant, the overall relationships may still be valuable to this research. Multiphysics COMSOL was applied to solve the flow governing. Physics-controlled mesh with fine size was utilized to generate the computational mesh.

In this study, the deposition is calculated as deposition fraction, which is defined as the percentage of the particles deposited in the olfactory region to the particles released into the nose. Here, if the particle moves back to the inlet it will disappear, if it reaches the olfactory region it will accumulate, and if it sticks to the other wall it will settle and freeze. In COMSOL, the particle deposition tracking simulation was based on the accumulator tool at the wall. In the accumulator, when a particle reached the target surface it was 100% absorbed and counted. The geometrical and physical properties of the aerosol particles are given in Table 1.

### 2.3. Correlation Analysis

It is of interest to identify the key acoustic parameters influencing the olfactory delivery efficiency. With this aim, cross correlation analyses of some acoustic parameter and olfactory deposition versus frequency domain were conducted [25]. In the statistical sciences, cross-correlation is a measure of the resemblance between two time series as a function of the displacement of one relative to the other. The cross-correlation is similar in nature to the convolution of two functions. In an autocorrelation, which is the cross-correlation of a signal with itself, there will always be a peak at a lag of zero, and its size will be the signal energy. Another measure used is dynamic time warping (DTW), an algorithm for measuring similarity between two temporal sequences, which may vary in speed [26]. In addition to a similarity measure between the two sequences, a so called “warping path” is produced, and by warping according to this path the two signals may be aligned in time. The signal with an original set of points X(original), Y(original) is transformed to X(warped), Y(warped).

## 3. Results

### 3.1. Grid Independence Study

Mesh sensitivity analysis was conducted by comparing the eigenfrequency among four mesh sizes, where the grid-independent result was attained as shown in Table 2. The percentage of relative error from the fine mesh value of final captured particles in the olfactory region of case 1 in the frequency of 545 (19.4) is reported in Table 2.

### 3.2. Acoustic Eigenfrequency Analysis

**Figure 3:**
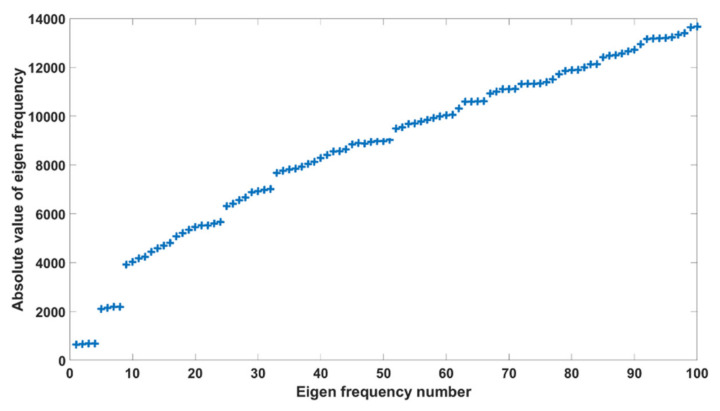
Eigenfrequency analysis.

Figure 3 shows the first one hundred eigenfrequencies of the four nasal airway configurations. The collection is made of four groups of the first 25 eigenfrequencies of each case. In the lower end, the eigenfrequency magnitudes are close to each other among the four geometries; the differences among geometries increase at higher eigenfrequency numbers (N). After the first two frequencies, which are around 668 and 2156 with less than 4% difference, the remaining frequencies could be fitted with a polynomial (−0.5 N^2^ + 153.9 N + 2621.7), with less than 7% relative error. The eigenfrequencies in Figure 3 are potential candidates for higher drug delivery to the olfactory region or other critical regions in the nose.

### 3.3. Olfactory Deposition with Acoustic Radiation Force

**Figure 4:**
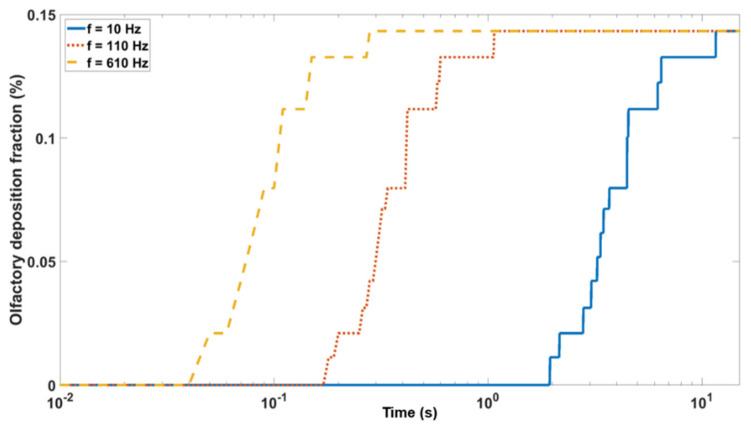
Predicted olfactory deposition at varying frequencies.

Figure 4 shows the deposition fraction in the olfactory region under three pulsating frequencies in Case 2 (baseline, with normal meatus dimensions). Increasing the pulsating frequency decreased the particle settling (i.e., the time to reach the steady-state deposition condition. The settling time for the frequency of 10 Hz, 110 Hz, and 610 Hz was 11.61 s, 1.07 s, and 0.28 s, respectively (Figure 4).

**Figure 5:**
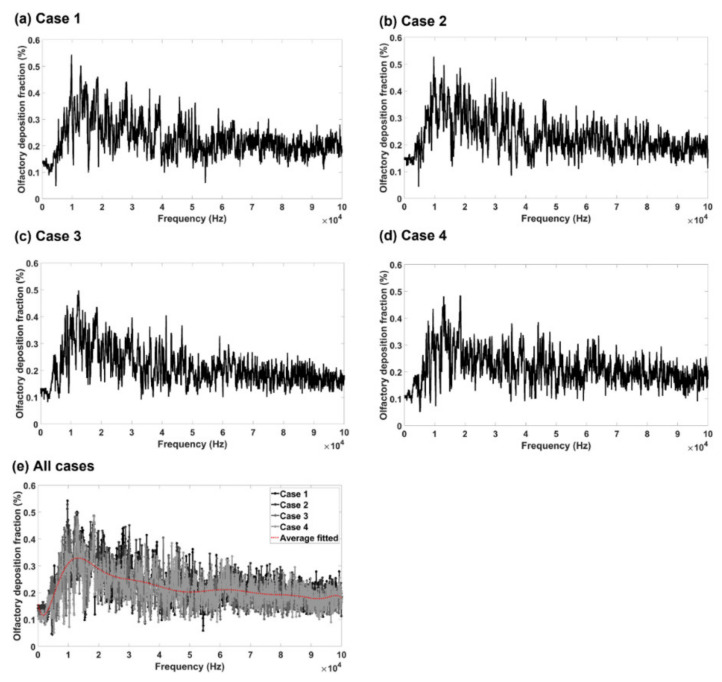
Comparison between obtained results of frequency analysis of (**a**) Case 1, (**b**) Case 2, (**c**) Case 3, (**d**) Case 4, and (**e**) all cases with an average line.

The olfactory deposition fraction vs. frequency in the range of 10–100,000 Hz is shown in Figure 5a–d for Cases 1, 2, 3, and 4, respectively, with a frequency increment of 50 Hz. Thus, the deposition profile in each figure was compiled from 2000 COMSOL files of numerical deposition simulations, whose size were around two terabytes. Despite the differences in peak values, a similar trend was observed among the four nasal geometries considered. In addition, turbinate shrinking/swelling did not have a significant effect on the magnitude of the olfactory delivery efficiency, while the optimal olfactory dose occurred at different frequencies for different nasal configurations.

To visualize the deposition trend more clearly, the four cases are plotted in Figure 5e together with an average line that describes the general trend of all cases. In most frequency ranges (i.e., 40–100 kHz), the averaged olfactory delivery efficiency did not change much with frequency. The optimal olfactory delivery was obtained within a limited range of 11–15 kHz.

**Figure 6:**
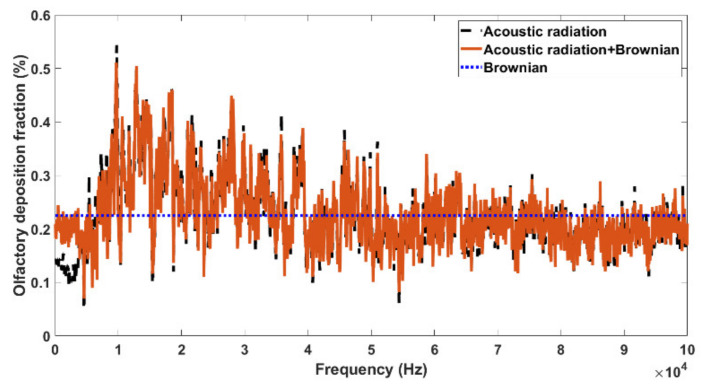
Effect of Brownian motion on olfactory deposition in the baseline case (Case 2).

Figure 6 shows the effect of the Brownian force on the olfactory deposition in comparison to that of the acoustic radiation force in Case 2 for 1 µm particles. As expected, a constant olfactory deposition fraction was predicted when only the random Brownian force was considered (blue line, Figure 6), which contrasted with the undulating profile under the acoustic radiation force. It was also observed that the Brownian force had a noticeable effect only in the low-frequency range (<5 kHz); for *f* > 5 Hz with the acoustic radiation force, there were negligible differences in the olfactory deposition with and without the Brownian force (red vs. black lines). Similar to Figure 5e, the optimal olfactory deposition with both the acoustic and Brownian forces was achieved around 10 kHz. Interestingly, the acoustic radiation force alone generated lower olfactory deposition than just the Brownian force when *f* < 5 Hz. However, when *f* > 40 Hz, the acoustic and Brownian forces alone led to equivalent olfactory deposition fractions (red vs. blue lines, Figure 6), indicating an increasing irregularity of the acoustic radiation force effects with increasing frequencies.

**Figure 7:**
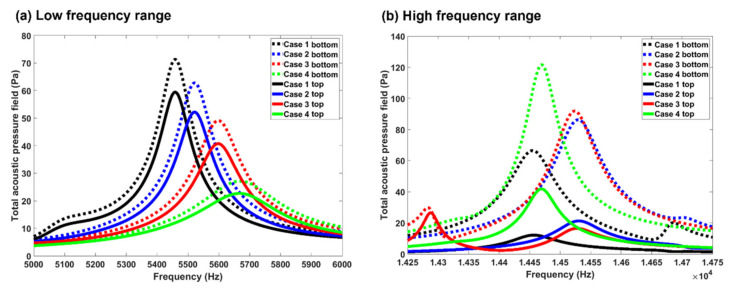
Comparison of acoustic pressure at some point at the top of olfactory volume and bottom of that region within the frequency range of (**a**) 5–6 kHz, and (**b**) 14,250 to 14,750 Hz.

Figure 7 compares the frequency response of the acoustic pressure in the four nasal geometries at two frequency ranges. In each geometry, the acoustic pressure at two sampling points was recorded, with one at the bottom of the olfactory region (dotted line) and the other at the top of the olfactory region (solid line). By choosing two points in the olfactory region, we seek to evaluate the change of the differential pressure vs. frequency.

As shown in Figure 7a, the differential pressure between these two probes pointed to the olfactory region and, therefore, would act favorably in dispensing particles to this region. As also noted in Figure 3, the eigenfrequencies in the range 5–6 kHz were found to be 5461, 5522, 5597, and 5667 Hz, respectively. Differing from Figure 3, the eigenfrequency analysis in Figure 7 was conducted with a refined frequency resolution (i.e., 1 Hz vs. 50 Hz in Figure 3). Considering the geometrical effects, increasing the turbinate cross-section decreased the magnitude of the acoustic pressure, indicating a weaker resonance in the nose with a larger turbinate. Moreover, increasing the turbinate cross-section decreased the differential pressure pointing to the olfactory region, thereby lowering the potential benefit to olfactory targeting. Figure 7b shows the frequency responses in the range of 14.25–14.75 kHz. In this case, the sequence of the pressure peaks had less correlation with the nasal geometry, while the magnitude of the differential pressure was relatively independent of the nasal geometry. The magnitudes of the differential pressure were much larger in the higher frequency range (~80 Pa) than in the low-frequency range (~10 Pa), and thus would lead to more improvements in the olfactory targeting.

**Figure 8:**
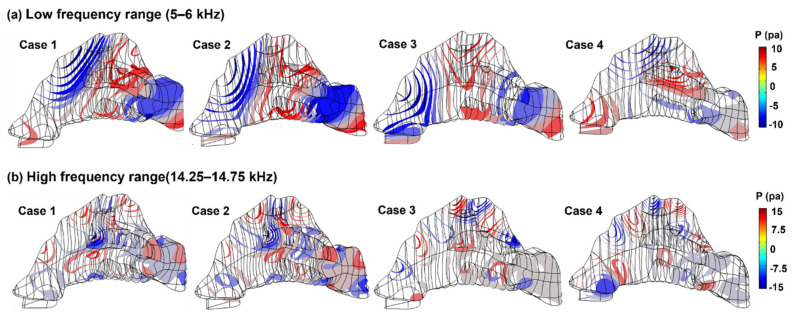
Comparison of acoustic pressure isosurfaces among four nasal geometries: (**a**) low-frequency range (5–6 kHz), and (**b**) high-frequency range (14.25–14.75 kHz).

To better understand the acoustic characteristics at varying eigenfrequencies, the pressure distributions inside the nasal cavity are plotted as iso-surfaces in Figure 8 for two frequency ranges considered (i.e., 5–6 kHz and 14.25–14.75 kHz). In the low-frequency range (Figure 8a), the iso-surfaces were more stratified, where the direction of the pressure gradient was more directed to the top of the nasal cavity than those in the high-frequency range (Figure 8b). Only one apparent pressure valley and one pressure peak were observed inside the nasal cavity for *f* = 5–6 kHz (Figure 8a). By contrast, there were multiple pressure valleys and peaks for *f* = 14.25–14.75 kHz (Figure 8b).

**Figure 9:**
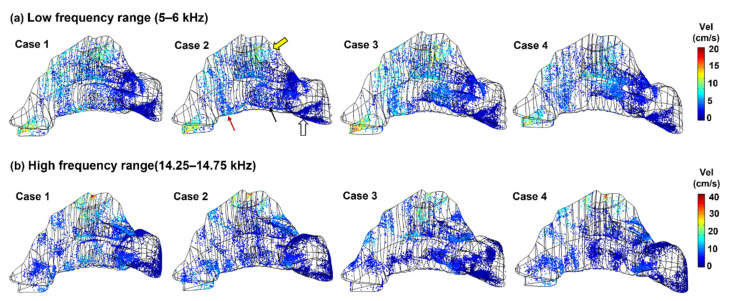
Comparison of particle surface deposition distribution among four nasal geometries: (**a**) low-frequency range (5–6 kHz), and (**b**) high-frequency range (14.25–14.75 kHz).

Figure 9 depicts the particle deposition distributions at low and high eigenfrequencies in the four nasal geometries. Highly heterogeneous deposition patterns were observed for both frequency ranges. When *f* = 5–6 kHz (Figure 9a), stripes of particle accumulations occurred in the nasal valve (red arrow), the inferior turbinate (black arrow), the nasopharynx (hollow arrow), and the upper turbinate (yellow hollow arrow), regardless of the nasal geometries. More concentrated particle accumulations occurred at the high eigenfrequency range 14.25–14.75 kHz, where high-dose patches were scattered throughout the nasal airway surface (Figure 9b).

**Figure 10:**
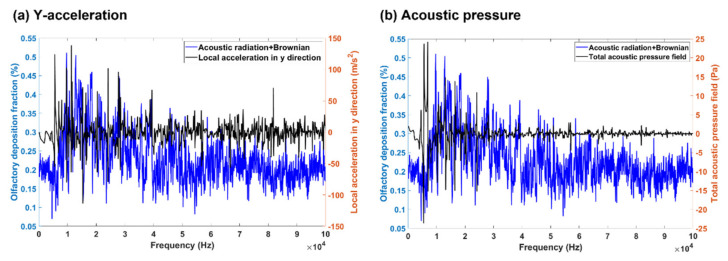
A general correlation between (**a**) acceleration in the y-direction, (**b**) acoustic pressure, and the olfactory deposition.

To identify the critical factors on olfactory drug delivery, various parameters were examined in terms of their cross-correlation with the olfactory deposition. Figure 10 shows the correlations of the olfactory deposition in the baseline case (Case 2) with the local acceleration in the y-direction (Figure 10a) and with the acoustic pressure (Figure 10b), respectively. In Figure 10a, the acceleration y component oscillates near a constant value; there were also several peaks, and one peak was coincident with the maximal olfactory deposition (*f* = 9750). Such behavior was expected because the olfactory mucosa was located in the maximum y location of the nose and the y-acceleration component helped transport particles to the olfactory region.

Figure 10b presents the correlation between the olfactory deposition and the acoustic pressure, which oscillated near a constant value. No significant correlation was observed between the peak of the acoustic pressure and the maximal olfactory deposition, indicating that the acoustic pressure was a weaker measure than the y-component of acceleration in determining the optimal olfactory delivery.

Other parameters were also evaluated to identify the key factors in olfactory drug delivery, which included the total acoustic pressure field, local velocity (RMS), instantaneous local acceleration, pressure, pressure times velocity, pressure times pressure gradient, and *x*, *z* components of the local acceleration. For the sake of conciseness, only the results of cross-correlation are presented in Table 3. The place of evaluation for each parameter is different. Some parameters were evaluated in the olfactory region (i.e., OL), and some were evaluated in the entire nasal cavity (i.e., by N). Surprisingly, the parameters evaluated in the entire nasal cavity had higher values of cross-correlation with the olfactory deposition, while parameters evaluated in the olfactory region had lower cross-correlation values (Table 3).

Various statistical measures were calculated for the parameters. Cross-correlation is generally used to measure the association between two different series. A higher cross-correlation value shows that the two sets are more likely identical. The first measure of the correlation used here is maximal cross-correlation (MCC). As shown in Table 3, the minimum cross-correlation was associated with the product of pressure and velocity, while the maximum cross-correlation was associated with the product of pressure and pressure gradient, which was the first term in acoustic radiation force (see Equations (3) and (4)). Another index that was used to evaluate the parameter was the peak difference (PD). PD measures the frequency distance between the maximum frequency of the parameter and those of olfactory deposition. This feature can use to estimate the saturation value of the olfactory absorption graph (see Figure 5e). The nearest peaks occurred at the *y* component of local acceleration, which was the direction pointing to the olfactory region. The comparison of these two signals is illustrated in Figure 10a. In the Cartesian coordinate system used here, *z* was the general direction of flow in longitudinal direction of the nasal pathway, *y* was the normal direction, and *x* is in the lateral direction of nose pathway. The last index used here was the unnormalized distance based on the Dynamic Time Warping Algorithm (DTWA), which is an algorithm for measuring the similarity between two time series. The minimum distance was found for the total acoustic pressure field while the maximum distance was for the *x* component of local acceleration. It means that regardless of peaks on the Figure or delay between signals the acoustic pressure has the same general manner of particle deposition.

## 4. Discussion

The results of this study suggested that the optimal frequency of sound waves emitted at the nostril for olfactory drug delivery was in the range 11–15 kHz. This result was compiled from more than 8000 numerical deposition test cases of 8 terabytes in COMSOL. Four nasal geometries were evaluated in a wide frequency spectrum of 10–100,000 Hz. The reasons for the optimal frequency being 11–15 kHz in olfactory targeting may be explained as follows. As illustrated in Figure 7a,b, valleys and peaks of the acoustic pressure exist inside the nasal cavity, which can act as a stabilizing or destabilizing force to collect or repel particles. In other words, the interactions between the nasal airway and input sound waves lead to either “friendly” zones, where the acoustic pressure gradients point to the olfactory region and help drive particles toward it, or “unfriendly” zones where the acoustic pressure gradients push particles away from the olfactory region. In Figure 7a (*f* = 5–6 kHz), a pressure valley (i.e., friendly zone) was observed that covered the upper-front nose and part of the olfactory region, with particles in the covered regions being more likely to deposit in the olfactory region. At higher frequencies (14.25–14.75 kHz, Figure 7b), the acoustic pressure valleys/peaks are smaller and distributed in an alternating manner. An “unfriendly“ zone in the proximity of the olfactory region can effectively block particles from reaching it. As a result, the total area of the “friendly” zones to the olfactory region did not constantly increase with the frequency but rather reached a peak around 11–15 kHz and decreased slightly at higher frequencies.

The predicted olfactory deposition fractions in this study (i.e., 0.6%) were lower than those in many previous studies. For instance, Schroeter et al. [27] measured the nasal deposition of 10.3 µm particles and reported a maximum olfactory deposition fraction of 5%. However, it is noted that the olfactory dosing depends on the defined region that was designated as “olfactory region”. In Schroeter et al. [27], the olfactory region included both the superior turbinate and the olfactory region. More importantly, local particle deposition in the nasal cavity is highly sensitive to the location of the nose due to its convoluted structure. Only a small fraction of drug particles can escape the filtration by the nasal valve and reach the turbinate; among them, even a much smaller fraction can change direction from the axial direction to move upward and reach the increasingly narrowed olfactory region. Xi et al. [22] compared the olfactory delivery efficiencies based on different definitions of the olfactory region and noted that the difference could be as large as one order of magnitude. As a result, a comparison of the olfactory deposition fractions among different studies should include how the targeted olfactory region was defined. Furthermore, the low olfactory deposition rates predicted in this study highlight the challenges of N2B drug delivery and call for more studies to investigate new delivery strategies to dispense drugs to this region.

There are several assumptions that may limit the applicability of the results of this study. First, the inhalation flow was not considered, and the results of this study can only represent the scenarios of breath-holding or very slow breathing conditions. Second, only four nasal geometries were considered, which cannot represent the intra-subject or inter-subject variability. Moreover, three nasal geometries were generated by modifying a patient-specific nose model. More patient-specific models are needed to verify the generality of the results in this study. Third, fluid-structure interactions, as well as the nasal wall compliance, were neglected. Moreover, there were no benchmark experiments yet to validate the olfactory deposition under radiation forces. Future in vitro studies using 3D printed nasal casts can provide valuable data to improve the accuracy of the numerical model developed in this study. After saying this, the predominant physics (i.e., acoustic-particle-wall interactions) were considered here in anatomically accurate nasal geometries under acoustic radiation forces of a wide spectrum (*f* = 10–100,000 Hz). In doing so, extensive computing was conducted, and deposition test cases with eight terabytes in size were compiled to identify the optimal frequency range for olfactory drug delivery. The results of this study can provide guidance to future studies of acoustic-aided intranasal drug delivery to the olfactory region.

## 5. Conclusions

A COMSOL-based computational model for acoustic-aided olfactory drug delivery was developed in four nasal geometries. A systemic parametric study of the acoustophoretic guidance to the olfactory region was performed for frequencies of 10–100,000 Hz at a resolution of 50 Hz. Findings were extracted from multi-terabyte computational results that could be used as guidelines for future acoustic-aided intranasal olfactory drug delivery. The major findings are:

(1) Frequency analyses of the nasal cavity revealed that certain eigenfrequencies were particularly associated with the olfactory region and had the potential to deliver more drugs to the olfactory region.
(2) At high frequencies, the acoustic pressure peaks/valleys were smaller in size and distributed in an alternating manner.
(3) Turbinate shrinking/swelling did not have a significant impact on the magnitude of the olfactory delivery efficiency but would alter the frequency for optimal olfactory delivery.
(4) The olfactory deposition was maximal at 11–15 kHz and decreased at higher frequencies. The olfactory deposition was almost constant for *f* > 40 kHz.
(5) A higher cross-correlation between the product of pressure and pressure gradient and the olfactory deposition delivery efficiency was found than other acoustic parameters. The nearest peaks belong to the y component of local acceleration, and the minimum unnormalized distance based on dynamic time warping algorithm belongs to the total acoustic pressure field.
(6) Frequency analysis near the olfactory region showed a negative correlation between acoustic pressure and turbinate cross-section.

## Figures and Tables

**Table 1:** Geometrical and physical constant values of parameters used in Equations (1)–(4).

	Value
$c_{c}$	343 [m/s]
T	293.15 [K]
p_A_	1 [atm]
$\rho_{c}$	1.24 [kg/m^3^]
$\rho_{p}$	1000 [kg/m^3^]
$d_{p}$	1 [μm]
$K_{p}$	2.2 [GPa]

**Table 2:** Grid independent study: comparison between various numerical solutions.

	Fine	Normal	Coarse	Coarser
Number of edge elements	5930	4254	2855	3397
Number of boundary elements	18,888	10,396	4484	6370
Number of elements	40,743	19,631	3652	11,343
Minimum element quality	0.01095	0.03195	0.01201	0.009745
Number of vertex elements	1057	1057	1057	1057
Error (%)	0	1.66	5.09	26.30

**Table 3:** Cross-correlation between various parameters and the olfactory deposition.

Parameter	Place	MCC	PD	DTWA
Total acoustic pressure field	OL *	358.3626	2850	0.0330
Local velocity (RMS)	OL	76.6382	6650	0.0484
Instantaneous local acceleration	OL	111.6919	65,500	0.0694
Pressure	OL	370.0679	4150	0.0334
Pressure × velocity	OL	13.1760	6650	0.0688
Local acceleration, y component	OL	307.4364	1600	0.0700
Pressure × pressure gradient	OL	329.0383	14,400	0.0694
Total acoustic pressure field	N **	539.3815	14,400	0.0694
Local acceleration, *x* component	N	238.5432	9700	0.5424
Local acceleration, *y* component	N	306.3583	14,400	0.0695
Local acceleration, *z* component	N	399.8635	72,100	0.0696

* Zone of averaging is the olfactory region. ** Zone of averaging is all domains of the nasal geometry.

## Data Availability

The data that support the findings of this study are available from the authors, upon reasonable request.
